# Effects of intensive home visiting programs for older people with poor health status: A systematic review

**DOI:** 10.1186/1472-6963-8-74

**Published:** 2008-04-03

**Authors:** Ans Bouman, Erik van Rossum, Patricia Nelemans, Gertrudis IJM Kempen, Paul Knipschild

**Affiliations:** 1 Department of Epidemiology, Faculty of Health, Medicine and Life Sciences, Maastricht University, Peter Debyeplein 1, 6229 HA Maastricht, The Netherlands; 2 School for Public Health and Primary Care (Caphri), Maastricht University, Maastricht, The Netherlands; 3 Department of General Practice, Maastricht University, Maastricht, The Netherlands; 4 Centre on Autonomy and Participation of the chronically ill, Zuyd University, Heerlen, The Netherlands

## Abstract

**Background:**

Home visiting programs have been developed aimed at improving the health and independent functioning of older people. Also, they intend to reduce hospital and nursing home admission and associated cost. A substantial number of studies have examined the effects of preventive home visiting programs on older people living in the community; the findings have been inconsistent. The objective of this review was to assess the effectiveness of intensive home visiting programs targeting older people with poor health or otherwise with functional impairments.

**Methods:**

A search for literature was based on included trials from four reviews on the effectiveness of home visits published after 2000 and on a database search of Cinahl, the Cochrane Central Register of Controlled Trials, Embase, Medline and PsycINFO from 2001 onwards. We also manually searched reference lists from potentially relevant papers. Randomized controlled trials were included assessing the effectiveness of intervention programs consisting of at least four home visits per year, an intervention duration of 12 months or more, and targeting older people (aged 65 years and over) with poor health. Two reviewers independently abstracted data from full papers on program characteristics and outcome measures; they also evaluated the methodological quality.

**Results:**

The search identified 844 abstracts; eight papers met the inclusion criteria. Seven trials were of sufficient methodological quality; none of the trials showed a significant favorable effect for the main analysis comparing the intervention group with the control group on mortality, health status, service use or cost. The inclusion of less-intensive intervention programs for frail older persons would not have exerted a great influence on the findings of our review.

**Conclusion:**

We conclude that home visiting programs appear not to be beneficial for older people with poor health within the health care setting of Western countries.

## Background

Home visiting programs have been developed aimed at improving the health and independent functioning of older people. Also, they intend to reduce hospital and nursing home admission and associated cost. A substantial number of studies have examined the effects of preventive home visiting programs on older people living in the community. Since 2000 four systematic reviews [1-4], a literature review [5] and a synthesis of several reviews [6] have been published. Furthermore, a review on the effectiveness of preventive primary care outreach interventions aimed at older people was published, but this review also included trials not based on home visiting programs [7]. The reviews produced inconsistent and conflicting results. Subgroup analyses of the largest meta-analysis showed that effective home-visiting programs include multidimensional assessment, many follow-up visits and targeted people at lower risk of death [3].

A trial in the Netherlands in the 1990s showed that home visits do not seem to be useful for the general population of older people, but subgroup analyses in this study suggested benefits for older people with poor health [8,9]. To investigate this further, a Dutch trial by Bouman *et al*. was recently conducted focusing on older people with poor health at baseline [10-13]. The study could not confirm beneficial effects on health status or service use for this target group. It is possible however that these findings constituted an isolated observation. In order to make a more well-founded judgment upon the effectiveness of home visits for this group of older persons, we decided to integrate the evidence from the study by Bouman *et al*. with evidence from other trials. Here we publish a systematic review to investigate whether the findings from this trial have also been reported by comparable studies targeting older people with poor health, or otherwise with functional impairments ('frail older persons'). Insight into whether home visiting programs for older people with poor health are effective is essential for implementation and future research. An assessment of the methodological quality of the included trials is also presented.

## Methods

### Search strategy

The search for trials until 2001 was based on the included trials from four systematic reviews (Table 1) [1-4]. These reviews consider a total of 30 trials. The systematic review by Ploeg *et al*. [7] was not included; none of the additional trials in this review were based on home visiting programs. Because the largest review and meta-analysis by Stuck *et al*. was based on an electronic search until 2001, we decided to continue our database search from this year onwards [3]. On July 11 2007, we searched the following databases: Cinahl, the Cochrane Central Register of Controlled Trials, Embase, Medline and PsycINFO. The following terms had to be used in the abstract or title: 'geriatric assessment', 'home visit*', 'health visit*', or 'health screening', in combination with 'prevent*' or 'screen*' (see Additional file 1). Because we were only interested in randomized controlled trials for an older population, we also included in the search the exploded medical subject heading (MeSH) terms 'randomized' and 'aged'; this restricted search with MeSH terms was, however, not possible within all databases. Reference lists from potentially relevant papers were manually searched for additional studies. A search for unpublished data was carried out for studies that had published a (design) article of their home visiting program, but without available outcome data or lacking other information necessary to determine eligibility. No language restrictions were imposed.

**Table 1:** Included trials in systematic reviews on home visiting programs

		Haastregt [1] 2000	Elkan [2]* 2001	Stuck [3] 2002	Meinck [4]^† ^2004
		Electronic search			

Author(s)	Year	1966–1999	1966–1997	1985–2001	up to 2003

Luker [48]	1981	x	x		
Gunner *et al*. [49]	1984			x	x
Vetter *et al*. [50]^‡^	1984	x	x	x	x
Hendriksen *et al*. [51]	1984	x	x	x	x
Sorensen *et al*. [52]	1988	x		x	x
Balaban *et al*. [33]	1988		x		x
Carpenter *et al*. [53]	1990	x		x	x
McEwan *et al*. [54]	1990	x	x	x	x
Oktay *et al*. [55]	1990		x		x
Clarke *et al*. [56]	1992			x	x
Hall [20]	1992	x	x		x
Pathy *et al*. [32]	1992	x	x	x	x
Hansen *et al*. [30]	1992		x		x
Williams et al. [27]	1992		x		x
Vetter *et al*. [57]	1992	x		x	x
van Rossum *et al*. [8, 9]	1993	x	x	x	x
Dunn *et al*. [34]	1994		x		x
Fabacher *et al*. [58]	1994	x	x	x	x
Tinetti *et al*. [29]	1994	x		x	x
Wagner *et al*. [59]	1994	x			x
Archbold *et al*. [60]	1995		x		x
Stuck *et al*. [61]	1995	x	x	x	x
Dalby *et al*. [21]	2000				x
Stuck *et al*. [15]	2000			x	x
van Haastregt *et al*. [23, 24]	2000			x	x
Hebert *et al*. [62]	2001			x	x
Newbury *et al*. [63]	2001			x	x
Gill *et al*. [44]	2003				x
Yamada *et al*. [22]	2003				x

*Evaluates home visiting programs that offer health promotion and preventive care; includes nonrandomized controlled trials.

† Based on the previous reviews with addition of three trials.

‡ One study describing two trials.

### Selection

We included randomized controlled trials examining the effects of home visiting programs for people aged 65 years and over. Based on earlier descriptions, preventive home visits are defined as visits to older people living in the community, which are aimed at multidimensional medical, functional, psychosocial, and environmental evaluation of their problems and resources [1,3,5]. This evaluation results in specific recommendations aimed at reducing or treating the observed problems and preventing new ones. Follow-up visits are included for the implementation of the intervention plan.

The target populations were older people with a poor health status based on either subjective (e.g. self-rated health) or more 'objective' measures (e.g. (self-reported) functional impairments, and dependencies in (instrumental) activities of daily living). It has been suggested that higher risk older persons would benefit most from a more-intensive intervention that includes systematic follow-up and coordination as well as more frequent visits [14-16]. If we were to expect benefits for this target population, a more intensive intervention may be a necessary requirement. We therefore decided to include studies with a relatively long and intensive follow-up, that is, when the intervention programs consisted of at least four home visits per year and the duration of the follow-up home visit period lasted 12 months or more; the home visits were to be carried out by health professionals, e.g. nurses or general practitioners. Since we focused on older people with poor health, who mostly suffer from multiple health problems, we excluded studies targeted at people with one specific disease. Studies without available data on health status, service use or cost were also excluded.

### Validity assessment

The quality of the research methods was evaluated using an adaptation of the Cochrane Back Review Group list of criteria [17]. Four items were omitted, because these were used as inclusion criteria (random allocation and relevance of outcome measure) or were not applicable to the evaluated interventions (blinding of the participants and care provider). The criteria list consists of five descriptive, two statistical, and eight validity items. Each item was scored '+' if the criterion was fulfilled, '-' if the criterion was not fulfilled, and '?' if the information was not provided or was unclear. Scores on validity items ranged from 0 to 8 per trial. Trials with at least four fulfilled validity items were considered to be of 'sufficient methodological quality' [18]; only those were included in the evaluation of the effectiveness. The items were scored independently by two reviewers (AB and PN). Disagreement was resolved by consensus or a third party (EvR).

### Data abstraction

Titles and abstracts resulting from the database search were independently screened (AB and PK). Full papers were obtained for potentially relevant studies. Data from all relevant papers were independently abstracted (AB and PN); this included data on characteristics of the home visiting programs and outcomes of the trials. Qualitative data abstraction was performed because of heterogeneity between trials regarding interventions and outcome measures. If information was absent from the original paper, attempts were undertaken to obtain complete information from the authors. Reviewers were not blinded to authors' names or institutions or journal of publication. Disagreement was resolved by consensus or a third party (EvR).

## Results

### Trial flow

**Figure 1:**
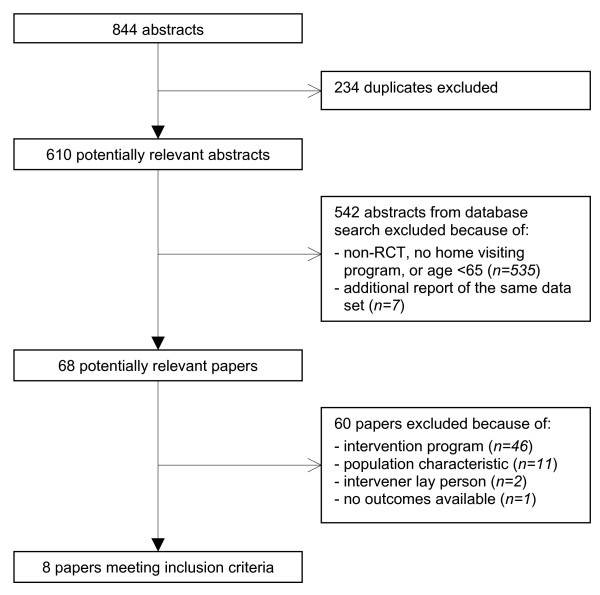
Progress of search for relevant trials.

Eight hundred and forty-four abstracts were identified, of which 234 duplicates were discarded (Figure 1). After screening whether the papers referred to randomized controlled trials that investigated home visiting programs for populations aged 65 years and over, we excluded another 542 abstracts. The remaining potentially relevant papers (n = 68) were further screened for population and intervention characteristics. Most papers were excluded because the intervention did not consist of at least four visits per year or the duration of the intervention period was less than 12 months (n = 46). Another 14 papers were excluded because the target population did not consist of older people with poor health (n = 11), the interveners were volunteers (n = 2), or there were no outcomes available (n = 1). Eight trials met the inclusion criteria; six of these have already been described in the previous systematic reviews and two studies were newly added. Design articles had been published of the two new studies; for the trial by van Hout *et al*. [19] no published outcomes were available and the outcomes from the trial by Bouman *et al*. [10] had been accepted for publication [12,13]. We contacted van Hout *et al*. for available data on the results of their study; we received information to complete Tables 2, 3 and 4 (see below), but they could not supply estimates of their data to complete Table 5 (see below), because those were due for publication elsewhere.

**Table 2:** Methodological quality of the trials meeting the inclusion criteria

	Hall [20]	Rossum [8]	Dalby [21]	Stuck [15]	Haastregt [23]	Yamada [22]	Hout [19]	Bouman [12]
*Descriptive items*								

1 Were eligibility criteria clearly specified	+	+	+	+	+	+	+	+
2 Were index and control interventions explicitly described	+	+	+	+	+	+	+	+
3 Was there a description of whether adverse effect had or had not occurred	-	+	+	+	+	+	+	+
4 Was a short-term follow-up measurement (directly after the intervention) performed	+	+	+	-	+	+	+	+
5 Was a long-term follow-up measurement (6+ months after the intervention) performed	-	-	-	+	+	-	-	+

*Statistical items*								

6 Was the sample size for each group described	+	+	+	+	+	+	+	+
7 Were point estimates and measures of variability presented	-	+	+	+	+	+	+	+

*Validity items*								

8 Was treatment allocation concealed	+	+	+	+	+	?	+	+
9 Were groups similar at baseline regarding age, sex, outcome	-	+	-	-	+	+	+	+
10 Were co-interventions avoided or comparable	?	?	?	?	?	?	?	?
11 Was compliance acceptable in all groups	?	+	?	?	+	+	?	+
12 Was the outcome assessor blinded to the intervention	+	+	+	+	+	+	+	+
13 Was the withdrawal/dropout rate acceptable (max of 20% for short-term follow-up and 30% for long-term follow-up)	-	+	+	+	+	+	-	+
14 Was timing of the outcome assessment in both groups comparable	+	+	+	+	+	+	+	+
15 Did the analysis include an intention-to-treat analysis	?	+	+	+	+	+	+	+
Sum score validity items								
+	3	7	5	5	7	6	5	7
?	3	1	2	2	1	2	2	1
-	2	0	1	1	0	0	1	0

*Notes*: scores +, criterion fulfilled; -, criterion not fulfilled; ?, data not provided or unclear (The results of the study by van Hout *et al*. have not been published yet; questions 3, 7, 9 and 13 were assessed from unpublished information; questions 10 and 11 could not be assessed.)

**Table 3:** Characteristics of the included home visiting programs

Author(s) Year Country	Sample size nr I/C	Health status participants	Mean age	Intervention program*	Number of visits per year	Duration of intervention in years	Intervener	Compliance
Dalby [21] 2000 Canada	73/69	self-reported functional impairment, or admission to hospital or bereavement in the previous 6 months	79	multidimensional assessment; a care plan was developed together with the primary care physician	as needed (mean 18.9 hours)	1.2	primary care nurse	not reported
Stuck [15] 2000 Switzerland	116/231	high-risk status based on six baseline predictors of functional deterioration	82^†^	annual multidimensional assessment (with physical examinations); preventive home visits in collaboration with the project team's geriatricians	4 (mean 7.5^†^)	2	trained public health nurse	not reported
van Haastregt [23, 24] 2000 Netherlands	159/157	moderate impairments in mobility, score ≥ 3 on mobility control scale of the short-version sickness impact profile, or a history of recent falls (≥ 2 in previous 6 months)	77	multidimensional assessment with checklists and use of guidelines; systematic home visits	5 (mean 4.5)	1	trained community health nurse	46% for referrals and advice
Yamada [22] 2003 Japan	184/184	dependent in IADL, independent in ADL, and not rating their health as excellent	79	multidimensional assessment based on the MDS-HC; scheduled home visits, primary objective human interaction	4 (mean 5.1)	1.5	trained public health nurse	47% for advice
van Hout [19] 2005 Netherlands	331/320	self-reported health score in the worst quartile of at least two of six COOP-WONCA charts	≥ 75	multidimensional assessment with RAI-HC; systematic home visits, an individual care plan was set up complying with patient priorities together with the primary care practice	5	1.5	trained home nurse	not (yet) reported
Bouman [10,11] 2007 Netherlands	160/170	self-reported poor health status at baseline, score 1–5 on a scale from 1–10 (very poor-excellent health)	76	multidimensional assessment with EasyCare questionnaire and checklists; systematic home visits, individual plan in agreement with the older persons	5.3 (mean 7.3)	1.5	trained home nurse	65% for referrals 58% for advice

*Notes*: I, intervention group; C, control group; ADL, activities of daily living; LTC, Long Term Care; MDS-HC, minimal data set home care; COOP-WONCA, COOP functional health assessment charts; RAI-HC, resident assessment inventory home care.

* The control group received usual care.

† Mean over entire group of high-risk and low-risk older persons.

**Table 4:** Effects of home visits on the main outcome measures of the included trials

Measures	Dalby [21]	Stuck [15]	van Haastregt [23,24]	Yamada [22]	van Hout [19]	Bouman [12,13]
Sample size, I/C	73/69	116/231	159/157	184/184	331/320	160/170
Followed up, I/C	59/54	82/188	120/115	160/149	215/209	139/154
Follow-up, months	14	36	12 and 18	18	18	18 and 24
Mortality	○	○	○	○	○	○
Health status			○	○	○	○
Functional status (ADL, IADL)		○	●*	○	○	○
Mental health			○	○	○	○
Social functioning			○		○	○
Hospital admission	○	○	○		○	○
Nursing home admission	○	○			○	○
Home for older persons					○	○
Medical specialist contacts	○	○	○			○
GP contacts	○	○	○			○
Home nursing care		○	○		○	○
Home help			○		○	○
Financial evaluation			○			○

*Notes*: I, intervention group; C, control group; ADL, activities of daily living; IADL, instrumental (household) activities of daily living.

○ = data available; no statistically significant favorable effect for the intervention group compared to the control group.

● = data available; favorable effect for the intervention group compared to the control group.

* Favorable effect for the intervention group at 12 months of follow-up (end of the intervention), but not at 18 months.

**Table 5:** Effects of home visits on outcome measures of the included trials for the intervention and control group

Author(s), year Country\I/C	Sample Size	Followed up	Follow-up months	Mortality %	Functional status % dependent		Hospital admissions*		Nursing home admission^†^	
					ADL	IADL	mean	mean days	% users	mean days
Dalby [21], 2000 Canada	73/69	59/54	14	10/4			0.4/0.3	19/11	0/1	
Stuck [15], 2000 Switzerland	116/231	82/188	36	29/18	39/38	61/63			27/14	
van Haastregt [23,24], 2000 Netherlands	159/157	120/115	18	6/9	33.1/31.5^‡^		0.5/0.6	7/8		
Yamada [22], 2003 Japan	184/184	160/149	18	6/8	67/65^§^					
Bouman [12,13], 2007 Netherlands	160/170	139/154	24	18/14	25/26	72/65	1.0/0.8	8/8	6/7	14/14

*Notes*: I, intervention group; C, control group; ADL, activities of daily living; IADL, instrumental (household) activities of daily living.

* Mean number of admissions and length of stay per person in the intervention and control group, respectively, during the follow-up period.

† Mean percentage of users and length of stay per person in the intervention and control group, respectively, during the follow-up period.

‡ Frenchay activities index (scores 13–52, highest score is most favorable).

§ Any problem in usual activities.

(The results from the study by van Hout *et al*. [19] have not been published yet; the estimates are not available.)

### Validity assessment

Table 2 presents the results of the methodological quality assessment of the eight trials meeting the inclusion criteria. The observed sum score on the validity items ranged from 3 to 7 points (out of range 0–8 points). Only the trial by Hall [20] did not fulfill at least four criteria and was considered to be of insufficient methodological quality; the other seven trials were of sufficient methodological quality. Data on whether co-interventions were avoided or comparable between the intervention and control group, or whether the compliance was acceptable, was often not provided. For three trials the study groups were not similar at baseline [15,20,21]. Most papers however reported a concealment of treatment allocation, blinding of the outcome assessor to the intervention and inclusion of an intention-to-treat analysis.

### Study characteristics

Characteristics of six home visiting programs are shown in Table 3. Two trials were not tabulated, because one was of insufficient methodological quality [20] and another presented only a subgroup with poor health from the studied general population of older people [8,9]. The targeted populations were people aged 65 years and over with poor self-reported health (in the trials by van Hout *et al*. [19] and Bouman *et al*. [12,13]); with instrumental activities of daily living (IADL) dependencies (in the trial by Yamada *et al*. [22]); or otherwise with functional impairments (in the trials by Dalby *et al*. [21], Stuck *et al*. [15] and van Haastregt *et al*. [23,24]). The trial by Yamada *et al*. also included a subgroup of older people with poor self-reported health from the original population with IADL dependencies [22]; this subgroup is not tabulated. The mean age of the study populations was mostly between 75–79 years. In one trial, the sample size was relatively small with 73 intervention and 69 control participants [21]. Most trials offered four or five home visits per year, with an intervention duration varying between 12 and 36 months. The mean number of visits was 4.5 [25], 5.1 [22], 7.3 [11], 7.5 [15], and, on average, 18.9 hours (from personal communication with the first author) [21]. The visits were carried out by home or health nurses; in two trials the assessment was done in cooperation with a primary care physician [19,21] and in another trial with a geriatrician [15]. Compliance with the recommendations was reported in half of the trials, varying between 46% and 65%.

### Outcome measures

None of the trials with sufficient methodological quality showed a significant favorable effect for the main analysis comparing the intervention group with the control group on mortality, health status, service use or cost (Table 4) [12,13,15,19,21-24]. For two trials, follow-up of outcome measurements were available at the end of the intervention period and 6 months thereafter [12,23]. Van Haastregt *et al*. found a favorable effect for the intervention group compared to the control group on functional status at the end of the intervention period (12 months), but this effect had disappeared after 18 months of follow-up [23]. None of the other outcomes in this study showed a beneficial effect of the program at 12 or 18 months of follow-up. In the study by Bouman *et al*. none of the outcomes showed statistically significant differences between the intervention and control group for follow-up measurements at 18 months (the end of the intervention period) or at 24 months [12]. The study by Stuck *et al*. included a home visit follow-up period of 24 months, but the outcome follow-up was measured at 36 months; no differences in favor of the intervention group compared with the control group were demonstrated [15].

The post hoc subgroup analyses (analyses done after looking at the data) of the trial by van Rossum [8,9] yielded positive effects for intervention participants on several outcome measures, e.g. on health status, IADL, admissions to hospital and homes for older persons, and mortality; the post hoc subgroup analyses of Yamada *et al*. [22] showed favorable effects for activities of daily living (ADL) and mental health. The one trial of insufficient methodological quality reported a favorable effect for the intervention group compared to the control group on nursing home admissions (not tabulated) [20].

For the most commonly measured outcomes shown in Table 4 (functional status, hospital and nursing home admissions, and mortality), more detailed information is provided in Table 5. The data on health status is not shown in this table, because the outcomes were based on a variety of different measurement instruments and these could not be reduced to a common denominator. For patients in the intervention and control group, respectively, means or percentages are presented – with unfavorable outcomes on mortality, functional status, and hospital and nursing home admissions. No further quantitative data synthesis or pooling was carried out because of the limited number of studies.

## Discussion

Of the eight randomized controlled trials evaluating the effectiveness of intensive home visiting programs for older people with poor health or otherwise with functional impairments, seven were of sufficient methodological quality according to the predefined standard. Even though nearly all studies were methodologically sound, improvements are still possible in specifying whether co-interventions were avoided or comparable between the intervention and control group, and, for half of the studies, in reporting the compliance with the interventions. The main analysis results from the trials of sufficient methodological quality consistently showed that the home visiting programs had no effect on the health status or service use of older people with poor health. Based on the information provided in these studies, also no differences were found between the intervention and control group in mortality.

In our evaluation, we did not include the results from the trial of insufficient methodological quality [20] and from post hoc subgroup analyses within two trials [8,22]. These studies reported some positive effects, but the results were based on small sample sizes, and, in the trial of insufficient methodological quality, the outcomes may also have been affected by the large drop-out rate and the dissimilarity of the study groups at baseline. The positive effects from the post hoc subgroup comparison from the earlier Dutch study [8] that indicated the visits to be effective for those with a poor perceived health status at baseline could not be confirmed by the results from a larger replication study [12].

The review consists largely of published information; some unpublished data was obtained from two systematically searched studies [19,21]. We did not further supplement the database search with expert consultations and it is possible that unpublished data from other trials is available. Studies with significant, positive, results are generally easier to find than those with non-significant or negative results and this could lead to a bias toward a more positive result [26]. Since nearly all studies in our review have negative results, publication bias can not explain the results. To ensure an acceptable standard for design and quality, only randomized controlled trials were included and an assessment of validity items was provided. It is rather striking that the one trial, that provided positive results, was considered to be of poor methodological quality by both reviewers.

In the review by van Haastregt *et al*. [1] no clear evidence was found in favor of the effectiveness of preventive home visits to older persons. The review and meta-regression analysis by Stuck *et al*. [3], including 11/15 of the studies from van Haastregt *et al*. (see Table 1), also showed no effect on mortality, nursing home admissions or functional status. Both reviews included trials with different target populations and interventions. Meta-analytic subgroup analyses in the review by Stuck *et al*. suggested, however, benefits from home visiting programs for certain types of patients or interventions. For instance, when interventions were targeted at persons with a lower risk for death (defined as annual mortality rates between 3% and 6%), a reduction in functional decline was shown. No reductions were shown for target populations with a mortality rate above 6%. This latter finding is in agreement with the findings in this review, wherein mortality rates were above 6% in most studies. Meta-analyses in the review by Elkan *et al*. [2], including 8/15 studies from van Haastregt *et al*., also focused on subgroups of frail older persons: no effects were shown on functional status, but positive effects were shown on mortality and nursing home admissions. However, these findings for frail older persons were based on only four studies, including one nonrandomized controlled trial [27] and one we scored in our review as being of insufficient methodological quality [20].

From the search, a number of home visiting programs targeting frail older persons were not included in our review because the intervention consisted of less than four visits per year and/or the duration of the intervention program was shorter than 12 months. The results of studies on less-intensive interventions are mixed. Five studies out of nine showed positive results: two studies with benefits for the intervention group on fall-related outcomes [28,29]; one study showing more nursing home admissions in the control group [30]; one study showing improvements in psychosocial functioning for the intervention group [31]; and one study demonstrating a shorter length of stay at the hospital in the intervention group in the younger group (aged 65–74), but not in the older group (aged 75 or older), and better self-rated health scores in the intervention group (no baseline measurements were available) [32]. Four studies out of nine had negative results [33-36]. As most positive trials (5/9) showed benefits for only one parameter, while the other trials were negative (4/9), the overall benefits seem limited. In case we had used different inclusion criteria and added these studies, this would not have exerted a great influence on the findings of our review.

The mean number of visits of the trials presented in Table 3 ranged from 4.5 to 7.5, and, on average, 18.9 hours; for the non-included studies targeting frail older persons this ranged from 1 to approximately 3 [28,30,32-36], with the exception of 7.8 visits in the Tinetti *et al*. study [29] and a median of 5 visits in the Markle-Reid *et al*. study [31]. The last two studies were not included, because their intervention period lasted 6 months or less. In general, we succeeded in including studies with more-intensive programs. These did not, however, result in more favorable outcomes for the intervention group compared to the control group. Based on our definition of home visiting programs, all included trials reported a multidimensional assessment with follow-up, also with physical examination in the study by Stuck *et al*. [15]. Of the less-intensive non-included programs for frail older persons, Hebert *et al*. [36] and Tinetti *et al*. [29] included a multidimensional assessment with physical examination. There seemed no apparent relationship between characteristics of the assessments and beneficial effects of the programs.

There is still debate on the frailty concept. The definition and identification of frail older persons varies considerably [37,38]. As there is no consensus yet, we used 'poor health status' as inclusion criteria, but we also referred to 'frail older persons'. Because of the wide use in defining frail populations, we decided that the inclusion could be based on either subjective measures (e.g. self-rated health, which is an overall measure for functional health abilities, including physical, mental and social functioning and has shown predictive validity for mortality and institutionalization among older persons [9,39]) or on more 'objective' measures (e.g. ADL-scores). Also other measures, e.g. 'at risk for falls' or 'functional impairments', have been described for target populations of frail older persons, and these studies were eligible. The non-included home visiting studies also targeted frail older persons, but did not match our criteria on the intervention program.

The results of the trial by Bouman *et al*. in the Netherlands have been supported by all methodologically sound studies evaluated in this review [12]. The results from the post hoc subgroup analysis from an earlier Dutch study on which this trial was based, could not be confirmed [8]. The CONSORT statement [40] already made notice of the risk of spurious findings from subgroup analyses [41,42], and indicated that especially post hoc subgroup comparisons are likely not to be confirmed by further studies. This is in line with the findings of this review. Results from reported post hoc subgroup analyses should therefore be interpreted with great caution.

Contrary to our expectation, the findings of this review suggest that intensive home visiting programs targeted at older people with poor health status are not effective. As half of the included studies were carried out in the Netherlands and the other half in other Western countries, the results may only be applicable in comparable health care settings. There seem to be no arguments to add the home visiting program targeting older persons with poor health status to regular healthcare. In a number of countries, e.g. Japan, Denmark and Australia, preventive home visits are part of the national policy and the visits are incorporated into regular healthcare for older persons. It seems essential, that the programs are judged on their merit again when targeting older persons in poor health. The United Kingdom withdrew this policy in 2004 based on the results of a large national trial, which showed that different forms of multidimensional assessment, targeting either the general population of older persons or frail older persons, offered almost no differences in patient outcomes [35].

Frail older persons may benefit from other types of interventions. In a recent editorial by Stuck and Kane, the authors indicated that older persons at higher risk or those already disabled are likely to benefit from multidimensional interventions that target specific problems [43], e.g. favorable effects were shown by a 6-month intensive home-based physical therapy program (16 visits over 6 months) [44], a chronic disease self-management program including three visits and nine telephone calls [45], and by a nurse-centered discharge plan with follow-up home visits for frail older persons discharged from hospital [46]. The economic arguments for these studies remain to be established however. Collaboration between different professionals involved may be necessary to manage the complex care that is often required by frail older persons. In a large European study (11 countries) it was found that home care service based on a case management approach reduced risk of institutionalization in a population of frail older persons in home care [47]. Although care-coordination can be provided by a case-manager, ideally the health and social services should be more integrated and have coordination between the services to supply the best available options for the individual needs of older persons.

## Conclusion

In conclusion, we think that intensive home visiting programs are probably not beneficial for frail older persons within the health care setting of Western countries. Since many older adults prefer to live in their own homes and the population of older adults, including frail adults, is expected to grow, future research is needed to search for alternative approaches to improve the health status of frail older persons.

## Competing interests

The author(s) declare that they have no competing interests.

## Authors' contributions

EvR and PK developed the original idea. AB performed the search strategy, abstracted data, and drafted the manuscript; PN abstracted data. PK and EvR assisted in the data abstraction process. EvR, GK, PK and PN provided valuable comments during the process of writing this manuscript. All authors read and approved the final manuscript.

## Pre-publication history

The pre-publication history for this paper can be accessed here:



## Supplementary Material

Additional file 1 Search strategies used. Searches used in the databases of Cinahl, Medline, PsycINFO and Embase. Click here for file
