# Supplementary material for: Effects of intensive home visiting programs for older people with poor health status: A systematic review
Source: BMC Health Serv Res. 2008 Apr 3;8:74. doi: 10.1186/1472-6963-8-74 (PMC2364620; doi:10.1186/1472-6963-8-74)
Supplement: Additional file 1 — Search strategies used. Searches used in the databases of Cinahl, Medline, PsycINFO and Embase. [file 1472-6963-8-74-S1.doc]

**Searches via the interface of the Maastricht University, Maastricht, The Netherlands**

**Cinahl**

Search History

#7 ((prevent* or screen* or (health education) or (health promotion) or (random*)) and (PY > 2001)) and (#2 and ((random* in AB) or (random* in TI)) and (PY > 2001)) and (PY > 2001)(254 records)

Searches and results below from: multiple databases

#6 ((prevent* or screen* or (health education) or (health promotion) or (random*)) and (PY > 2001)) and (#2 and ((random* in AB) or (random* in TI)) and (PY > 2001)) and (PY > 2001)(45 records)

Searches and results below from: multiple databases

#5 ((prevent* or screen* or (health education) or (health promotion) or (random*)) and (PY > 2001)) and (#2 and ((random* in AB) or (random* in TI)) and (PY > 2001)) and (PY > 2001)(363 records)

#4 (prevent* or screen* or (health education) or (health promotion) or (random*)) and (PY > 2001)(592411 records)

#3 #2 and ((random* in AB) or (random* in TI)) and (PY > 2001)(363 records)

#2 #1 and ((older or elderly) in AB) and (PY > 2001)(3060 records)

#1 (((geriatric assessment)or( home visit* )or( health visit* )or( health screening )) and (PY > 2001)) and (PY > 2001)(6948 records)

The search: ((prevent* or screen* or (health education) or (health promotion) or (random*)) and (PY > 2001)) and (#2 and ((random* in AB) or (random* in TI)) and (PY > 2001)) and (PY > 2001) returned 254 records

**Medline**

Search History

#5 ((prevent* or screen* or (health education) or (health promotion) or (random*)) and (PY > 2001)) and (#2 and ((random* in AB) or (random* in TI)) and (PY > 2001)) and (PY > 2001)(363 records)

#4 (prevent* or screen* or (health education) or (health promotion) or (random*)) and (PY > 2001)(592411 records)

#3 #2 and ((random* in AB) or (random* in TI)) and (PY > 2001)(363 records)

#2 #1 and ((older or elderly) in AB) and (PY > 2001)(3060 records)

#1 (((geriatric assessment)or( home visit* )or( health visit* )or( health screening )) and (PY > 2001)) and (PY > 2001)(6948 records)

The search: ((prevent* or screen* or (health education) or (health promotion) or (random*)) and (PY > 2001)) and (#2 and ((random* in AB) or (random* in TI)) and (PY > 2001)) and (PY > 2001) returned 363 records

**PsycINFO**

Search History

#6 ((prevent* or screen* or (health education) or (health promotion) or (random*)) and (PY > 2001)) and (#2 and ((random* in AB) or (random* in TI)) and (PY > 2001)) and (PY > 2001)(45 records)

Searches and results below from: multiple databases

#5 ((prevent* or screen* or (health education) or (health promotion) or (random*)) and (PY > 2001)) and (#2 and ((random* in AB) or (random* in TI)) and (PY > 2001)) and (PY > 2001)(363 records)

#4 (prevent* or screen* or (health education) or (health promotion) or (random*)) and (PY > 2001)(592411 records)

#3 #2 and ((random* in AB) or (random* in TI)) and (PY > 2001)(363 records)

#2 #1 and ((older or elderly) in AB) and (PY > 2001)(3060 records)

#1 (((geriatric assessment)or( home visit* )or( health visit* )or( health screening )) and (PY > 2001)) and (PY > 2001)(6948 records)

The search: ((prevent* or screen* or (health education) or (health promotion) or (random*)) and (PY > 2001)) and (#2 and ((random* in AB) or (random* in TI)) and (PY > 2001)) and (PY > 2001) returned 45 records

**Embase**

Search History

#8 ((prevent* or screen* or (health education) or (health promotion) or (random*)) and (PY > 2001)) and (#2 and ((random* in AB) or (random* in TI)) and (PY > 2001)) and (PY > 2001)(104 records)

Searches and results below from: multiple databases

#7 ((prevent* or screen* or (health education) or (health promotion) or (random*)) and (PY > 2001)) and (#2 and ((random* in AB) or (random* in TI)) and (PY > 2001)) and (PY > 2001)(254 records)

Searches and results below from: multiple databases

#6 ((prevent* or screen* or (health education) or (health promotion) or (random*)) and (PY > 2001)) and (#2 and ((random* in AB) or (random* in TI)) and (PY > 2001)) and (PY > 2001)(45 records)

Searches and results below from: multiple databases

#5 ((prevent* or screen* or (health education) or (health promotion) or (random*)) and (PY > 2001)) and (#2 and ((random* in AB) or (random* in TI)) and (PY > 2001)) and (PY > 2001)(363 records)

#4 (prevent* or screen* or (health education) or (health promotion) or (random*)) and (PY > 2001)(592411 records)

#3 #2 and ((random* in AB) or (random* in TI)) and (PY > 2001)(363 records)

#2 #1 and ((older or elderly) in AB) and (PY > 2001)(3060 records)

#1 (((geriatric assessment)or( home visit* )or( health visit* )or( health screening )) and (PY > 2001)) and (PY > 2001)(6948 records)

The search: ((prevent* or screen* or (health education) or (health promotion) or (random*)) and (PY > 2001)) and (#2 and ((random* in AB) or (random* in TI)) and (PY > 2001)) and (PY > 2001) returned 104 records

**Embase (RTC/aged exploded)**

Search History

#15 ((explode "randomized-controlled-trial" / all SUBHEADINGS in DEM,DER,DRM,DRR) and (PY > 2001)) and ((((explode "home-for-the-aged" / all SUBHEADINGS in DEM,DER,DRM,DRR) or (explode "aged-" / all SUBHEADINGS in DEM,DER,DRM,DRR)) and (PY > 2001)) and ((((health screening and (PY > 2001)) or ((health adj visit*) and (PY > 2001)) or ((home adj visit*) and (PY > 2001)) or (geriatric assessment and (PY > 2001))) and (PY > 2001)) and ((prevent* or screen* or (health education) or (health promotion)) and (PY > 2001)) and (PY > 2001)) and (PY > 2001)) and (PY > 2001)(26 records)

#14 ((explode "randomized-controlled-trial" / all SUBHEADINGS in DEM,DER,DRM,DRR) and (PY > 2001)) and ((((explode "home-for-the-aged" / all SUBHEADINGS in DEM,DER,DRM,DRR) or (explode "aged-" / all SUBHEADINGS in DEM,DER,DRM,DRR)) and (PY > 2001)) and (((health screening and (PY > 2001)) or ((health adj visit*) and (PY > 2001)) or ((home adj visit*) and (PY > 2001)) or (geriatric assessment and (PY > 2001))) and (PY > 2001)) and (PY > 2001)) and (PY > 2001)(50 records)

#13 (explode "randomized-controlled-trial" / all SUBHEADINGS in DEM,DER,DRM,DRR) and (PY > 2001)(58250 records)

#12 (((explode "home-for-the-aged" / all SUBHEADINGS in DEM,DER,DRM,DRR) or (explode "aged-" / all SUBHEADINGS in DEM,DER,DRM,DRR)) and (PY > 2001)) and ((((health screening and (PY > 2001)) or ((health adj visit*) and (PY > 2001)) or ((home adj visit*) and (PY > 2001)) or (geriatric assessment and (PY > 2001))) and (PY > 2001)) and ((prevent* or screen* or (health education) or (health promotion)) and (PY > 2001)) and (PY > 2001)) and (PY > 2001)(328 records)

#11 (((explode "home-for-the-aged" / all SUBHEADINGS in DEM,DER,DRM,DRR) or (explode "aged-" / all SUBHEADINGS in DEM,DER,DRM,DRR)) and (PY > 2001)) and (((health screening and (PY > 2001)) or ((health adj visit*) and (PY > 2001)) or ((home adj visit*) and (PY > 2001)) or (geriatric assessment and (PY > 2001))) and (PY > 2001)) and (PY > 2001)(683 records)

#10 ((explode "home-for-the-aged" / all SUBHEADINGS in DEM,DER,DRM,DRR) or (explode "aged-" / all SUBHEADINGS in DEM,DER,DRM,DRR)) and (PY > 2001)(337694 records)

#9 (((clinical article) or(clinical study) or(clinical trial) or(controlled study) or(randomized controlled trial) or(major clinical study) or(double blind procedure) or(multicenter study) or(single blind procedure) or(phase 3 clinical trial) or(phase 4 clinical trial) or(crossover procedure) or(placebo*)) and (PY > 2001)) and ((((health screening and (PY > 2001)) or ((health adj visit*) and (PY > 2001)) or ((home adj visit*) and (PY > 2001)) or (geriatric assessment and (PY > 2001))) and (PY > 2001)) and ((prevent* or screen* or (health education) or (health promotion)) and (PY > 2001)) and (PY > 2001)) and (PY > 2001)(636 records)

#8 ((clinical article) or(clinical study) or(clinical trial) or(controlled study) or(randomized controlled trial) or(major clinical study) or(double blind procedure) or(multicenter study) or(single blind procedure) or(phase 3 clinical trial) or(phase 4 clinical trial) or(crossover procedure) or(placebo*)) and (PY > 2001)(1427631 records)

#7 (((health screening and (PY > 2001)) or ((health adj visit*) and (PY > 2001)) or ((home adj visit*) and (PY > 2001)) or (geriatric assessment and (PY > 2001))) and (PY > 2001)) and ((prevent* or screen* or (health education) or (health promotion)) and (PY > 2001)) and (PY > 2001)(933 records)

#6 ((health screening and (PY > 2001)) or ((health adj visit*) and (PY > 2001)) or ((home adj visit*) and (PY > 2001)) or (geriatric assessment and (PY > 2001))) and (PY > 2001)(1856 records)

#5 (prevent* or screen* or (health education) or (health promotion)) and (PY > 2001)(397978 records)

#4 health screening and (PY > 2001)(417 records)

#3 (health adj visit*) and (PY > 2001)(225 records)

#2 (home adj visit*) and (PY > 2001)(758 records)

#1 geriatric assessment and (PY > 2001)(481 records)

The search: ((explode "randomized-controlled-trial" / all SUBHEADINGS in DEM,DER,DRM,DRR) and (PY > 2001)) and ((((explode "home-for-the-aged" / all SUBHEADINGS in DEM,DER,DRM,DRR) or (explode "aged-" / all SUBHEADINGS in DEM,DER,DRM,DRR)) and (PY > 2001)) and (((health screening and (PY > 2001)) or ((health adj visit*) and (PY > 2001)) or ((home adj visit*) and (PY > 2001)) or (geriatric assessment and (PY > 2001))) and (PY > 2001)) and (PY > 2001)) and (PY > 2001) returned 50 records
